# Editorial: Extracellular vesicles in age-related neurodegenerative disease: Biological mechanisms, diagnostics, and therapeutics

**DOI:** 10.3389/fnagi.2022.1015985

**Published:** 2022-09-06

**Authors:** Nan Zhang, Marc L. Gordon

**Affiliations:** ^1^Department of Neurology, Tianjin Neurological Institute, Tianjin Medical University General Hospital, Tianjin, China; ^2^The Litwin-Zucker Research Center, Northwell Health, The Feinstein Institutes for Medical Research, Manhasset, NY, United States; ^3^Departments of Neurology and Psychiatry, Donald and Barbara Zucker School of Medicine at Hofstra-Northwell, Hofstra University, Hempstead, NY, United States

**Keywords:** extracellular vesicle, neurodegenerative disease, exosome, diagnosis, treatment

Neurodegenerative diseases are usually age-related and associated with one or more misfolded and aggregated proteins. Although amyloid and tau protein can be measured with PET imaging or in cerebrospinal fluid, most neurodegenerative proteinopathies cannot be detected *in vivo*. Extracellular vesicles (EVs) are nanosized particles that arise from a wide range of cells and contain molecular cargo, including a variety of proteins, messenger RNAs (mRNAs), and microRNAs (Shah et al., 2018). Recently, the identification of protein and genetic biomarkers contained in EVs, in particular neuronal- or glial-derived EVs, has elucidated biological mechanisms and facilitated diagnosis in some neurodegenerative diseases, such as Alzheimer's disease (AD), Parkinson's disease (PD), and amyotrophic lateral sclerosis (ALS). Furthermore, EVs have also been investigated as potential therapeutic agents or targets for neurodegenerative diseases, either using EVs loaded with a therapeutic cargo, or pharmacological modification of the release of EVs containing associated pathological proteins. Neuronal-derived EVs, in particular exosomes (EVs of endosomal origin), have also been isolated from peripheral blood, and analyzed for the expression of proteins, such as beta-amyloid (Aβ), tau, cellular survival factors, lysosomal proteins, insulin receptor substrate and synaptic proteins, in relation to the diagnosis, prognosis and treatment of neurodegenerative diseases, including AD, frontotemporal lobar degeneration, and PD (Goetzl et al., 2016; Athauda et al., 2019; Kapogiannis et al., 2019; Jia et al., 2021). In our previous studies, levels of TAR DNA binding protein of 43 kDa (TDP-43) (Zhang et al., 2020) and matrix metalloproteinase-9 (MMP-9) (Gu et al., 2020) in addition to Aβ42 and phosphorylated tau-181 in neuronal-derived EVs from plasma were found to be elevated in patients with amyloid PET supported AD.

In addition to neuronal-derived EVs, astrocyte-derived EVs have been also demonstrated to participate in the pathological process of AD, in particular targeting the neurovascular unit. González-Molina et al. isolated astrocyte-derived EVs from 3xTg-AD mice (a transgenic mouse model of AD) and postmortem brain samples of sporadic or familial patients with AD, and observed that these EVs affected cell components of the neurovascular unit, such as astrocytes, endothelial cells and neurons, and induced cytotoxicity and astrocyte hyperreactivity *in vivo*. Neurovascular unit disruption and dysregulation of cerebral blood flow have been recognized to play an important role in the progression of AD pathology even in the initial stage (Zhang et al., 2018). The findings of this study further suggested that astrocyte-derived EVs might mediate vascular deterioration in the human AD brain.

Moreover, RNAs, such as mRNA and non-coding RNA, were prominently loaded in EVs and involved in intercellular communication. Sproviero et al. found both an overlap and a difference in mRNA and long non-coding RNAs (lncRNA) regulation between large (100–1,000 nm) and small (30–150 nm) EVs derived from plasma of patients with various neurodegenerative diseases, such as AD, PD, frontotemporal dementia and ALS. Bioinformatics and pathways analyses indicated common transcriptomic profiling underlying neurodegenerative processes, although the specific RNA transcript signature for different diseases needs further investigation.

With respect to diagnosis, Utz et al. used flow cytometry to analyze microvesicles (EVs released from the cell membrane) in cerebrospinal fluid carrying total and phosphorylated tau, and synaptic-related proteins, and observed an elevation in the percentages of synaptophysin-bearing (but not tau-bearing) microvesicles in patients with AD compared with non-inflammatory neurological disease controls, classified according to the AT(N) biomarker system. This finding indicated that not only molecular concentration but also number or percentage of EVs carrying target molecules were potential biomarkers for disease diagnosis and activity.

In terms of treatment, Zhang et al. reported that cerebral endothelial cell-derived small EVs (exosomes) from aged diabetes mellitus rats inhibited neurogenesis, whereas those from adult healthy normal rats alleviated diabetes mellitus-impaired neurogenesis, cognitive function and cerebral vasculature. These therapeutic effects of cerebral endothelial cell-derived small EVs, which crossed the blood brain barrier and were internalized by neural stem cells in the neurogenic regions of the subventricular zone and the subgranular zone of the dentate gyrus, might be attributed to increases of miR-1 and -146a and reductions of their target genes, such as myeloid differentiation primary response gene 88 and thrombospondin 1.

Gao et al. reviewed the role of exosomes in the pathophysiology, diagnosis, prognosis, and treatment of some neurodegenerative diseases, including AD, PD, Huntington's disease, and ALS. Due to their peripheral availability and ability to cross the blood brain barrier, exosomes have the potential to be used both as diagnostic biomarkers as well as drug carriers for the treatment of neurodegenerative disease. However, as discussed in this review, therapeutic safety and technical issues present major challenges to the clinical application of exosomes.

Although there are several limitations for most previous and current studies, such as standardization of methodologies including isolation, characterization and classification of EVs, and small sample size in different cohorts, EVs hold great promise for understanding the biological processes, discovering diagnostic tools and developing therapeutic approaches in neurodegenerative diseases.

## Author contributions

NZ drafted the manuscript. MG revised the manuscript for important intellectual content. Both authors contributed to the article and approved the submitted version.

## Conflict of interest

The authors declare that the research was conducted in the absence of any commercial or financial relationships that could be construed as a potential conflict of interest.

## Publisher's note

All claims expressed in this article are solely those of the authors and do not necessarily represent those of their affiliated organizations, or those of the publisher, the editors and the reviewers. Any product that may be evaluated in this article, or claim that may be made by its manufacturer, is not guaranteed or endorsed by the publisher.
